# Acute Bony Bankart Injury With Glenoid Rim Fracture Treated by Arthroscopic Bankart Repair

**DOI:** 10.7759/cureus.64980

**Published:** 2024-07-20

**Authors:** Ishan Shevate, Vikram Reddy Cheemala, Ashwin Deshmukh, Rahul Salunkhe

**Affiliations:** 1 Orthopaedics, Dr. D. Y. Patil Medical College, Hospital and Research Centre, Pune, IND

**Keywords:** transosseous repair, recurrent shoulder dislocation, glenoid bone loss, arthroscopic shoulder surgery, arthroscopic bankart

## Abstract

A bony Bankart lesion is a torn labrocapsular complex with a glenoid rim fracture. In this case report, a patient with an acute bony Bankart injury presented with severe shoulder pain and limited range of motion following a road traffic accident. The injury was diagnosed through imaging studies and required arthroscopic bony Bankart repair. The post-surgery rehabilitation program restored the patient's shoulder mobility, strength, and stability, significantly improving pain relief and functional ability. Overall, the case report highlights the importance of prompt diagnosis and appropriate surgical intervention in acute bony Bankart injuries, followed by a well-structured rehabilitation program to achieve optimal outcomes in pain relief, range of motion, and functional ability.

## Introduction

Bony Bankart injuries are a type of acute shoulder injury characterized by a labroligamentous complex with a fracture of the anterior glenoid rim and associated dislocation or subluxation of the shoulder joint [1]. Acute bony Bankart injuries typically occur as a result of high-energy trauma, such as sports-related injuries or motor vehicle accidents [2]. Twenty-three percent of Bankart cases are presented with bony Bankart lesions [3]. These injuries can lead to significant shoulder pain, instability, and functional impairment in the non-operative group compared to the operative group [4,5]. Open surgical fixations lead to larger incisions and dissection, rupture of the subscapularis, restriction of external rotation movements, and post-capsulorrhaphy surgery-induced arthropathy [6]. Arthroscopic surgery has been preferred over open Bankart surgery over the past two decades, particularly for minimal glenoid and humeral bone loss, due to its minimally invasive approach [7]. Here, we discuss a patient with an acute bony Bankart injury and their clinical presentation, diagnostic evaluation, treatment approach to the lesion, surgical procedure, and outcomes.

## Case presentation

**Figure 1:**
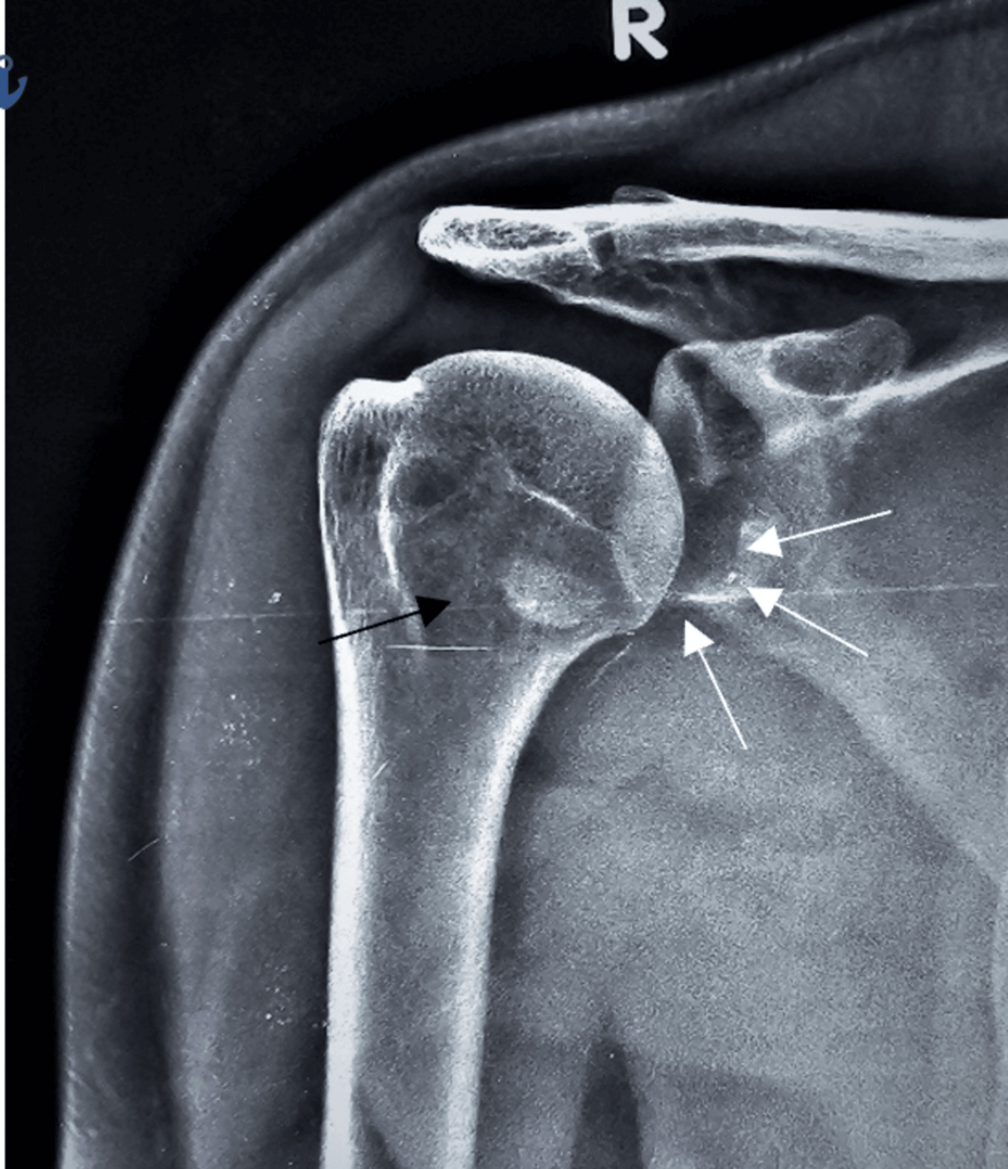
X-ray shows an anterior glenoid rim fracture (indicated by white arrow marks) with a free chondral fragment (shown in the black arrow mark).

**Figure 2:**
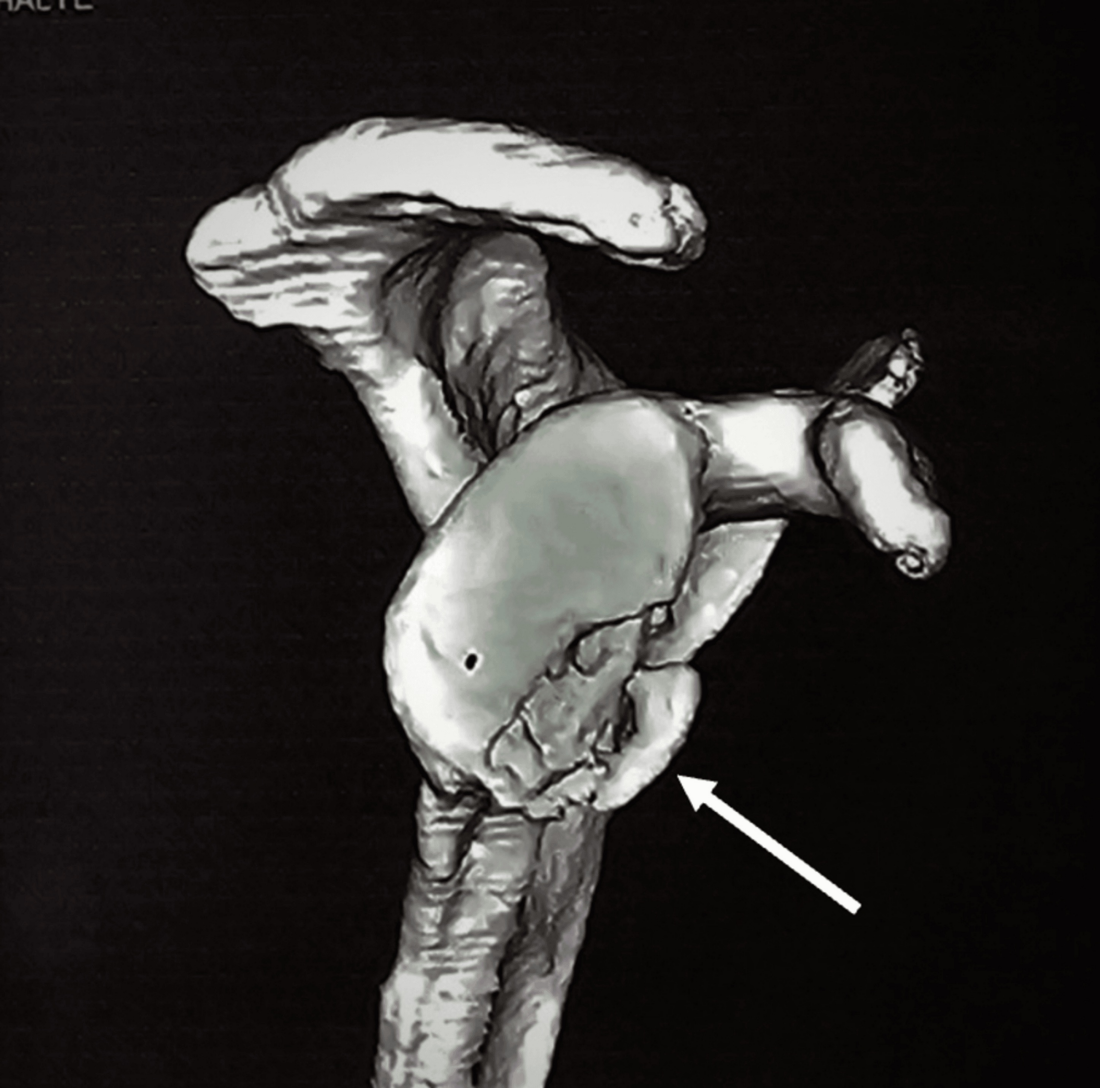
CT images showing the enface view of the scapula with an anterior inferior rim fracture of the glenoid with less than 15% bone loss with depressed osseous fragment (inidcated by the white arrow).

**Figure 3:**
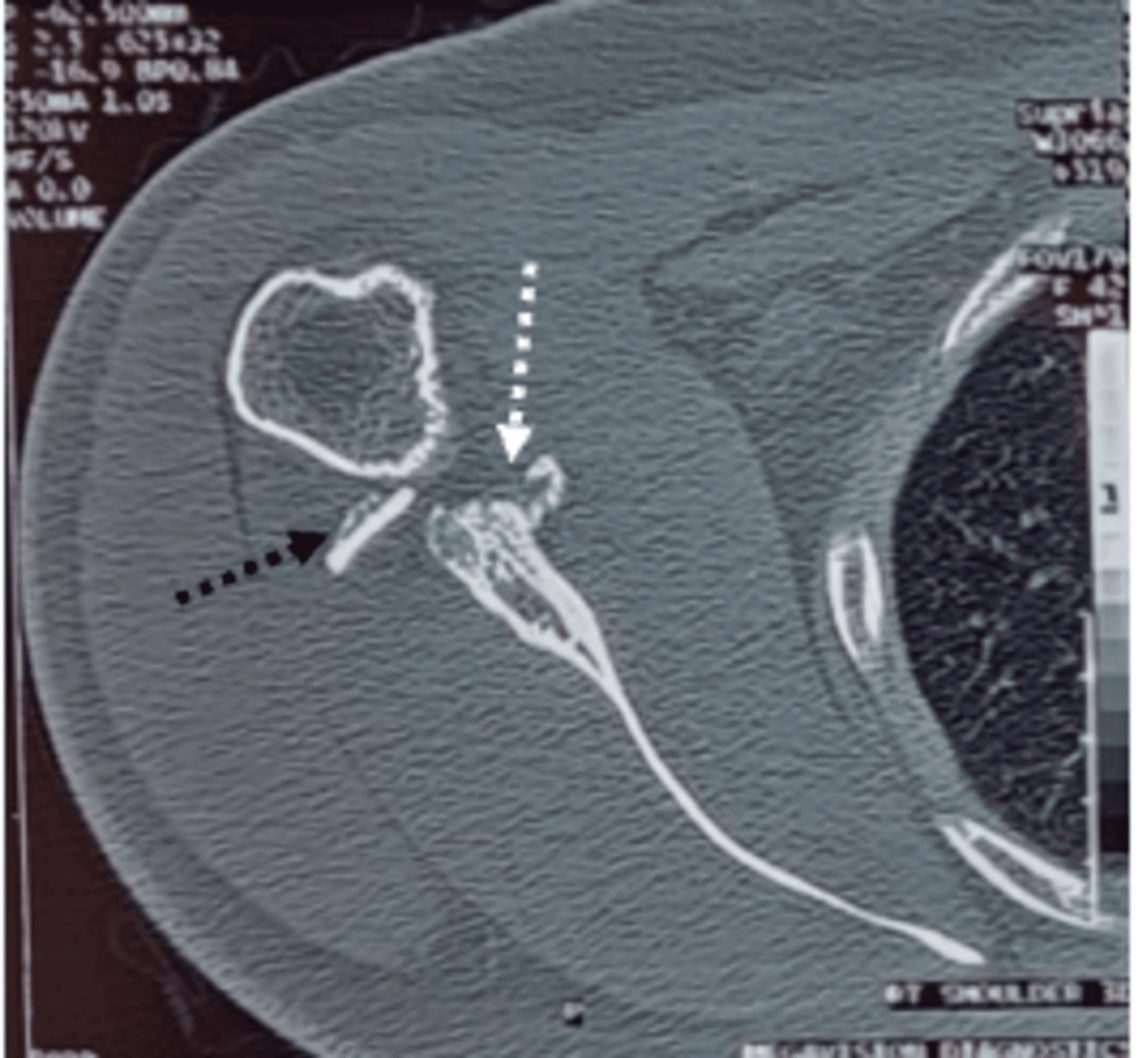
Free chondral fragment (indicated by the black dotted arrow) in the joint with a bony defect at the glenoid (indicated by the white dotted arrow).

**Figure 4:**
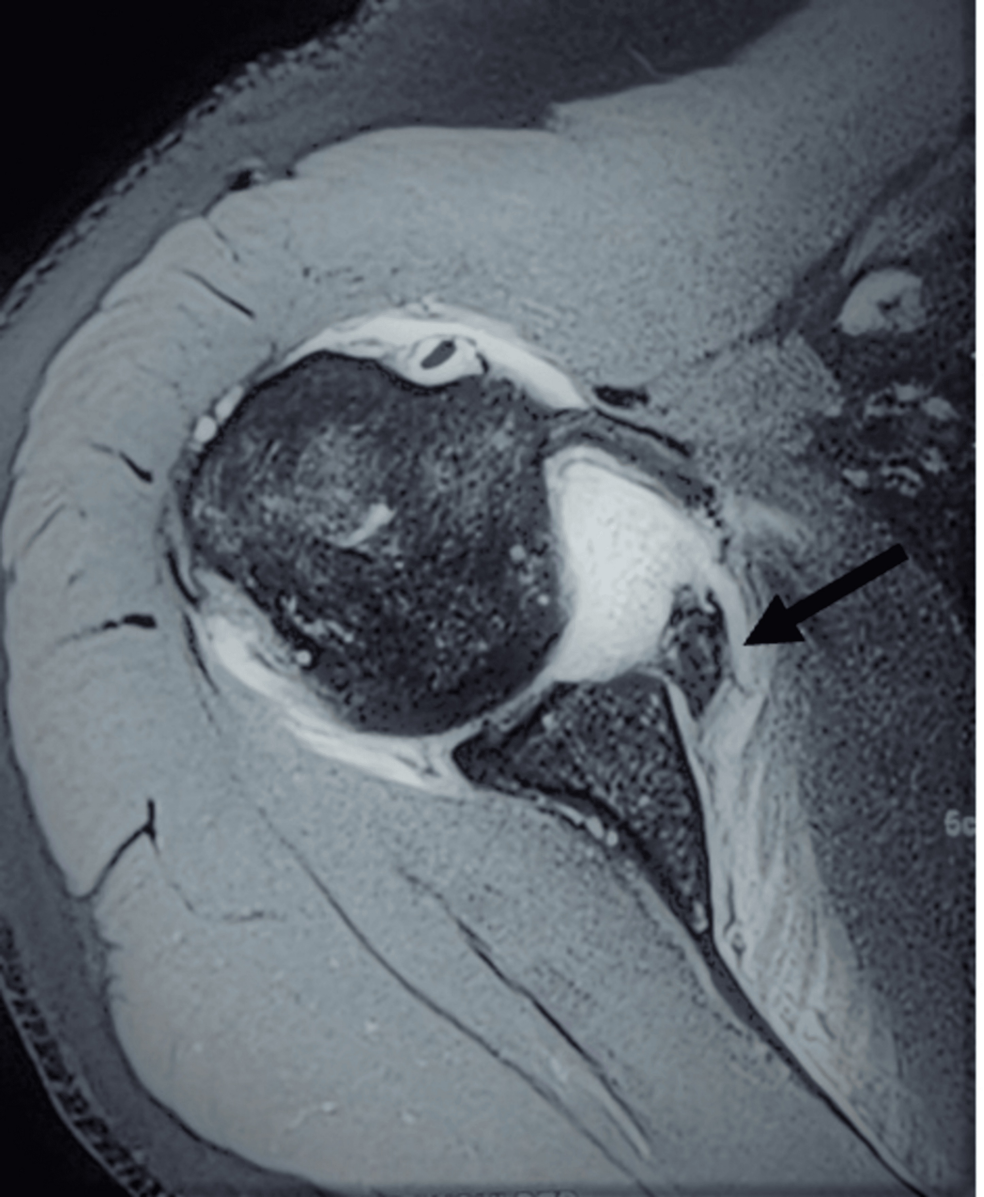
MRI showing a bony Bankart lesion with osseous fragment attaching to the labro capsular complex (indicated by the black arrow).

**Figure 5:**
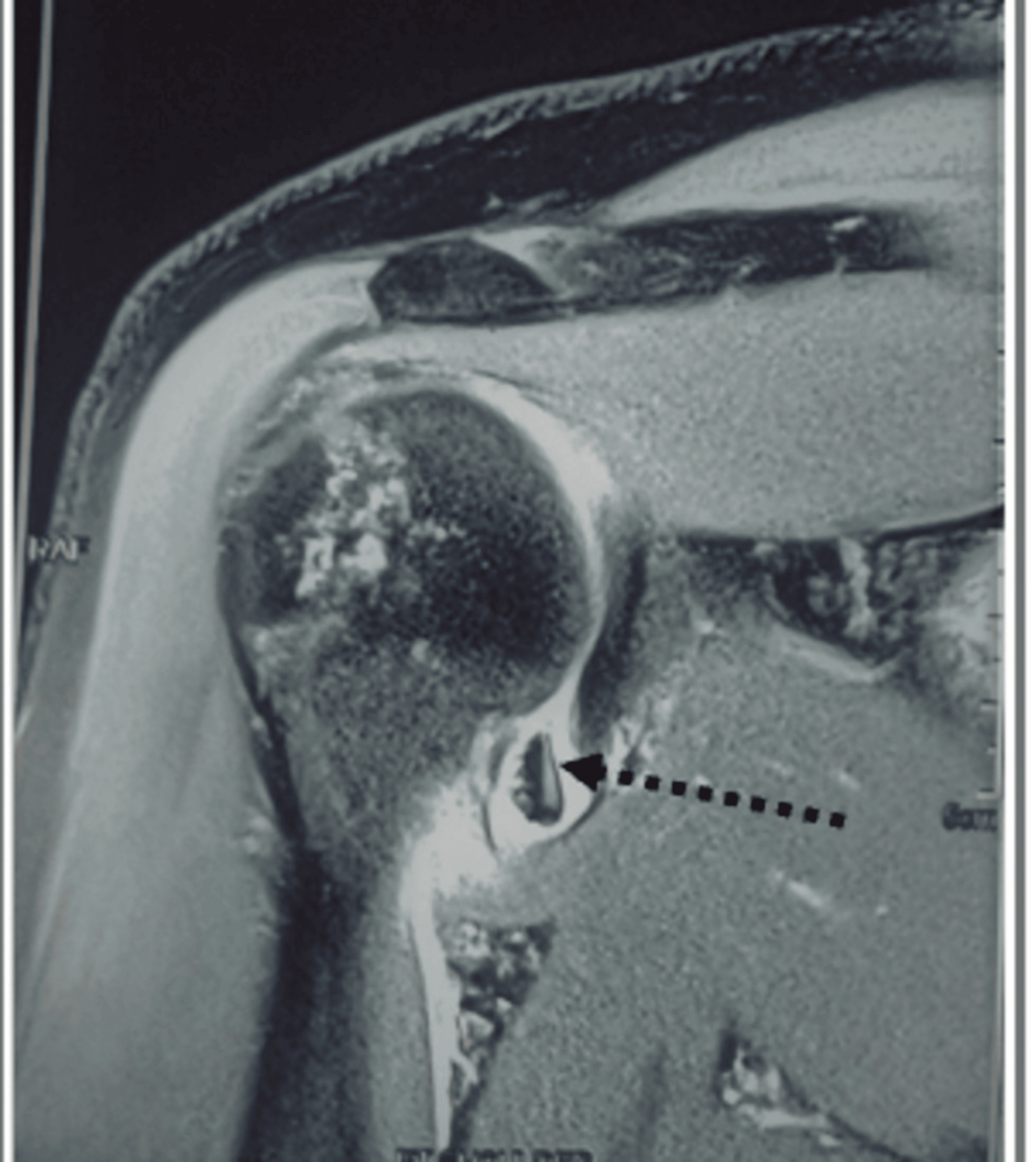
MRI coronal plane showing a free chondral fragment in the joint (black dotted arrow).

The case involves a 35-year-old male who presented to the emergency department with severe right shoulder pain, deformity, and restriction of movement following a road traffic accident. The patient reported a sudden pop in his shoulder during the fall. There was tenderness and swelling over the right shoulder upon examination, and the range of motion (ROM) was restricted. There were no signs of neurological deficits. Imaging studies, including X-rays and a CT scan, were performed to evaluate the extent of the injury. These imaging studies revealed a glenoid rim fracture (Figure 1) in the X-ray and CT scan, demonstrating a fracture of the anterior glenoid rim (Figure 2) and glenoid bone loss of less than 15% with a free articular chondral fragment (Figure 3). The patient was assessed for urgent care. Three days later, surgery was recommended based on magnetic resonance imaging (MRI) findings of a bony Bankart lesion (Figure 4, Figure 5). The patient underwent arthroscopic bony Bankart repair under general anaesthesia.

**Figure 6:**
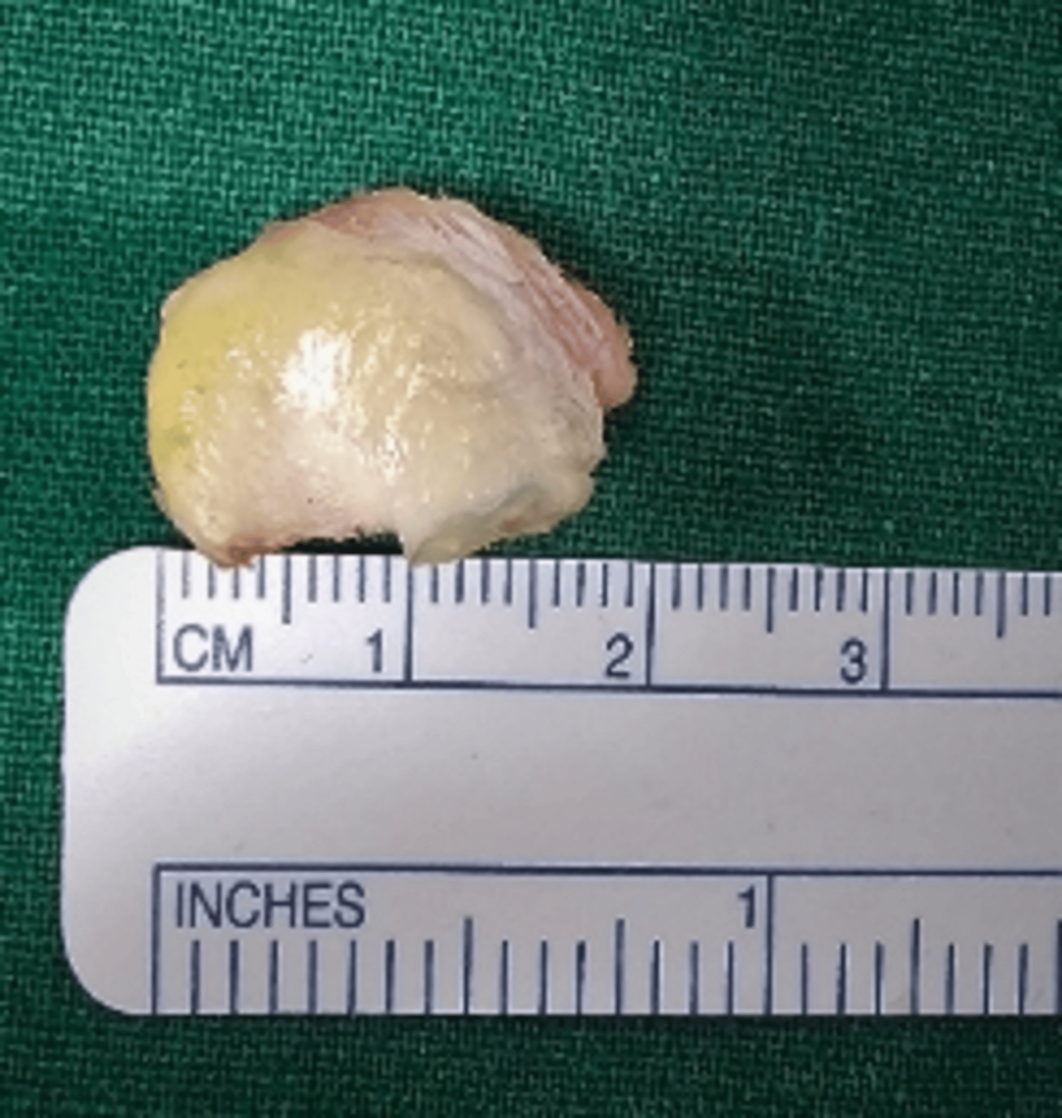
Free chondral fragment measuring 18 mm.

**Figure 7:**
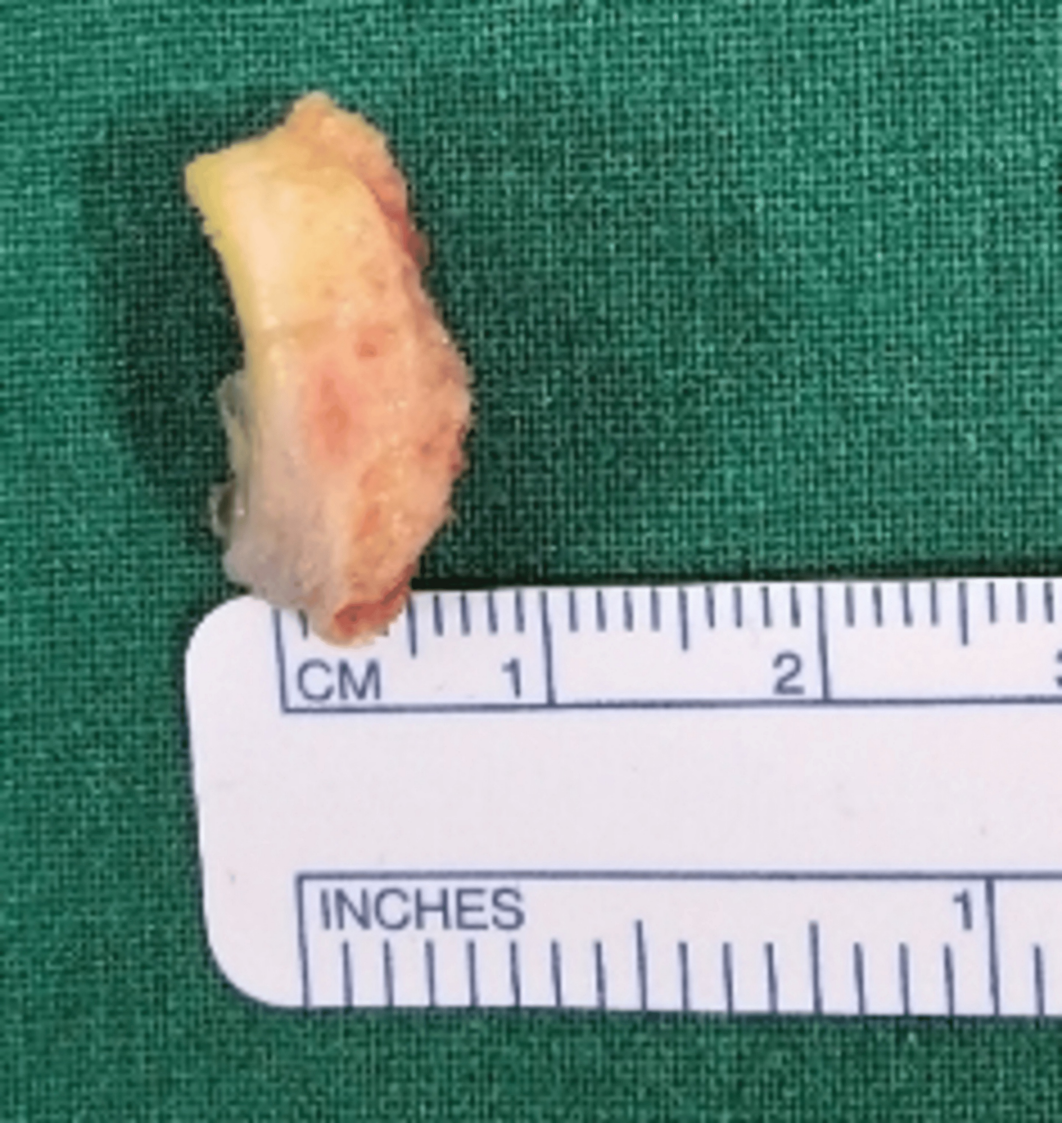
Free chondral fragment measuring a width of 5 mm.

**Figure 8:**
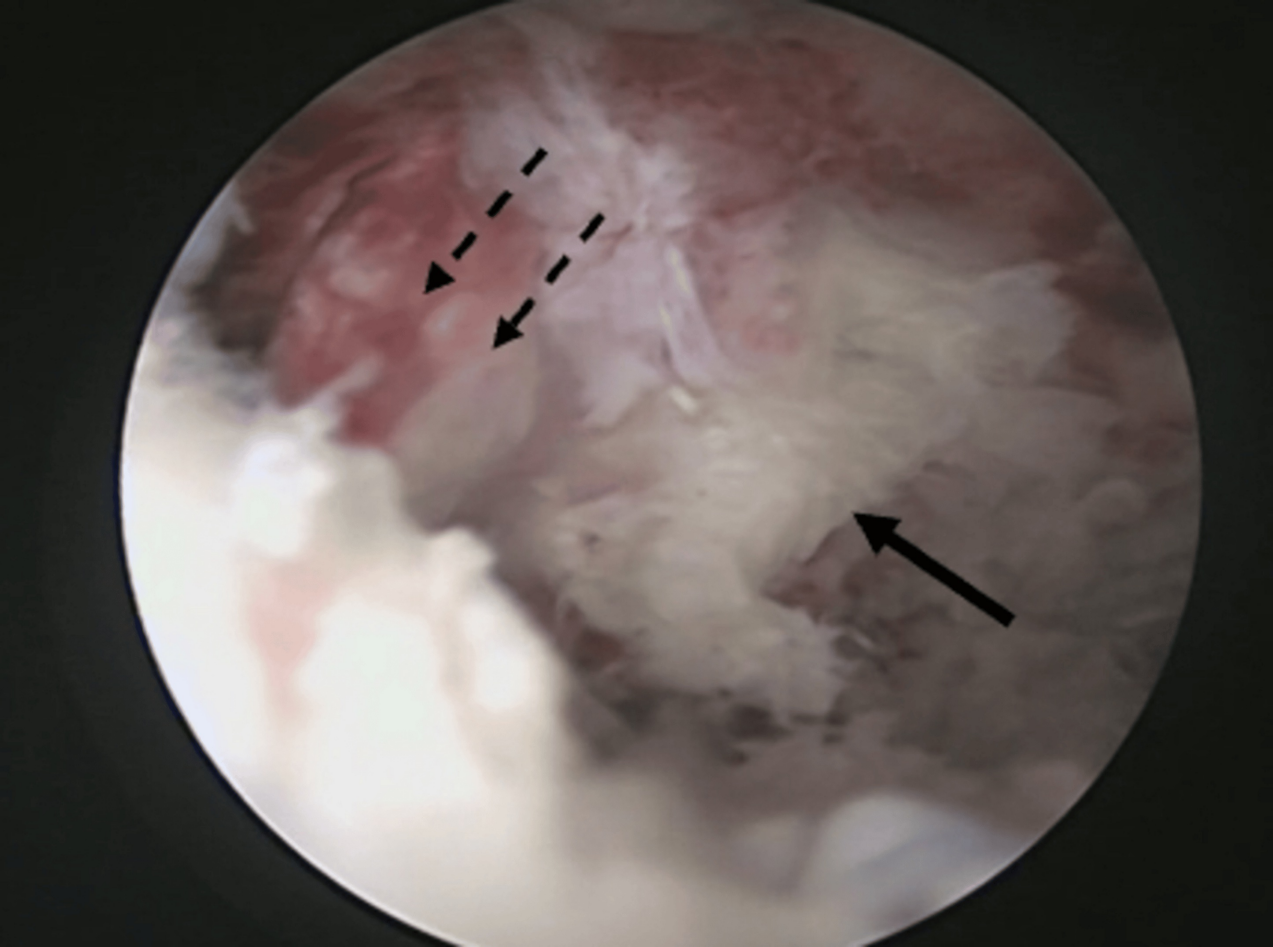
Liberated labrum (dotted black arrow) and osseous fragment (black arrow) from the glenoid neck.

**Figure 9:**
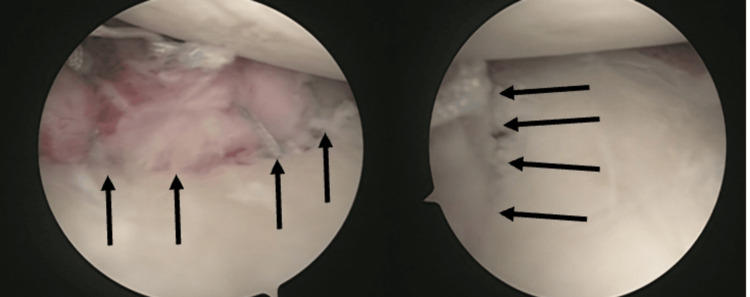
Reduced bony Bankart lesion with four suture anchors in place (black arrows).

Under general anaesthesia, the patient was placed in a lateral decubitus position with a 70-degree abduction and counterweight traction. During surgery, standard posterior viewing and anterior working portals were made, and the mobility of the fragment and extent of the labro capsular lesion were evaluated. An anterosuperior portal was placed for shuttling the sutures, posterior to the long head of the biceps in the rotator cuff interval. The free chondral fragment in the joint was removed, measuring about 18 x 5 mm (Figure 6, Figure 7). The anterior labrocapsular complex was torn from the 3 o'clock position to the 6 o'clock position with a bony defect of the glenoid and an osseous fragment attached to the labrum. The labrocapsular complex and the bony fragment were elevated (Figure 8) from the neck of the glenoid by a tissue liberator and radiofrequency ablator. The torn labrum was repaired using two 1.7 all suture anchors and two 2.4 PEEK single-loaded suture anchors by using the transosseous technique at 3, 4, 4.30, and 5 o'clock positions over the glenoid face by passing the sutures around the torn labrum and through the osseous fragment. The osseous fragment was reduced and held in place along with the torn labrum in its position (Figure 9). The stability of the glenohumeral joint was achieved, and wound closure was done.

The postoperative period was uneventful, and the patient's shoulder was immobilized for a month. After one month, the patient underwent a comprehensive rehabilitation program for shoulder mobility, strength, and stability. The rehabilitation program consisted of progressive exercises to improve ROM, strengthening exercises targeting the rotator cuff muscles, and proprioceptive training to enhance stability. The patient's progress was monitored through regular follow-up visits. At the six-month mark, the patient reported significant improvement in shoulder pain, increased ROM, and improved functional ability.

## Discussion

In acute bony Bankart injuries, 80-94% of patients had a recurrence of shoulder instability in young, sports participant patients who are managed conservatively and associated with difficulty in late surgical repair after failed conservative management. Over the years, acute bony Bankart lesions have been treated by open reduction and fixation. It is the go-to fixation for larger fragments of bony Bankart injuries [8]. In recent decades, there has been a shift to arthroscopic fixation procedures, and various techniques have been documented in the literature, but there is no one standard procedure for bony Bankart lesions. This is because there are several variables that affect the fixation techniques, including acute versus chronic, larger versus small fragments, age and activity level of the patient, technical skill, and surgeon’s preference [1]. One has to evaluate and plan majorly during the pre-operative period and if required intraoperatively. The size of the fragment is the detrimental factor that can be estimated by radiological and MRI imaging techniques [6].

A delayed surgical intervention can lead to an increased failure rate, anatomical injuries, and recurrent dislocations compared to early surgical interventions. Two dislocations before arthroscopic Bankart surgery more than doubled the chances of postoperative failure, according to Vaswani et al. [9]. Drain et al. stated that immediate surgical stabilization following a one-time dislocation significantly diminishes the risk of recurrent dislocation compared to those who undergo surgery following two dislocation events [10].

There are various arthroscopic intervention procedures for bony Bankart lesions postulated by various authors. Sugaya et al. liberated the displaced osseous fragment, which was firmly attached to the labroligamentous complex and was separated from the glenoid neck before reduction and fixation in the optimal position by suture anchors [11]. Braun et al. presented the “bony Bankart bridge procedure,” where a suture anchor is placed medially to the fracture on the glenoid neck and its sutures are passed around the bony fragment through the soft tissue, including the inferior glenohumeral ligament complex. The sutures are loaded and anchored into the glenoid face by the second anchor. This creates a non-tilting two-point fixation that compresses the fragment into its bed [12]. Field and Davis described three arthroscopic Bankart fixation techniques, i.e., labrum alone, transosseous, and double-row techniques, to address a range of presenting Bankart pathologies. In our case, we used the transosseous technique to fix the bony Bankart lesion that medialized the labrocapsular complex tear and fixed it to the glenoid at the chondral fragment avulsion.

## Conclusions

Acute bony Bankart lesions are not common injuries seen in routine practice. There is no single superior technique for managing acute bony Bankart lesions. The treatment approaches vary based on lesion characteristics and surgeon factors. Early diagnosis and management of such lesions with shoulder dislocation give better results by avoiding redislocation and early recovery of the patient. Arthroscopic repair of acute bony Bankart lesion requires pre-operative assessment of bony fragment and planning repair technique. Arthroscopic repair provides better results by early bony union and faster recovery due to its minimally invasive method. Post-surgery, monitored physiotherapy and tailored rehabilitation are essential for achieving optimal outcomes in such cases.
